# Assessment of household ownership of bed nets in areas with and without artemisinin resistance containment measures in Myanmar

**DOI:** 10.1186/s40249-018-0399-2

**Published:** 2018-03-23

**Authors:** Thae Maung Maung, Tin Oo, Khin Thet Wai, Thaung Hlaing, Philip Owiti, Binay Kumar, Hemant Deepak Shewade, Rony Zachariah, Aung Thi

**Affiliations:** 1 Department of Medical Research, Ministry of Health and Sports, Yangon, Myanmar; 2 Department of Public Health, Ministry of Health and Sports, Nay Pyi Taw, Myanmar; 3 Academic Model Providing Access to Healthcare (AMPATH), Eldoret, Kenya; 4 Gavi, the Vaccine Alliance, Geneva, Switzerland; 5 International Union Against Tuberculosis and Lung Disease (The Union), South-East Asia Regional Office, New Delhi, India; 6 Médecins Sans Frontières, Brussels Operational Centre, Luxembourg city, Luxembourg; 7 National Malaria Control Program, Ministry of Health and Sports, Nay Pyi Taw, Myanmar

**Keywords:** Malaria, Insecticide-treated bed nets, Long-lasting insecticidal nets, Myanmar artemisinin resistance containment, Bed nets ownership, Bed nets access, Bed nets utilization, Myanmar

## Abstract

**Background:**

Myanmar lies in the Greater Mekong Subregion where there is artemisinin-resistant *Plasmodium falciparum* malaria. As the artemisinin compound is the pillar of effective antimalarial therapies, containing the spread of artemisinin resistance is a national and global priority. The use of insecticide-treated bed nets/long-lasting insecticidal nets (ITNs/LLINs) is the key intervention for ensuring the reduction of malaria transmission and the spread of resistant strains, and for eventually eliminating malaria. This study aimed at assessing household ownership of, access to, and utilization of bed nets in areas of Myanmar with and without artemisinin resistance containment measures.

**Methods:**

Secondary data from a nationwide community-based malaria survey conducted by the National Malaria Control Program in 2014 were analyzed. Based on evidence of artemisinin resistance, Myanmar was divided into tiers 1, 2, and 3: townships in tiers 1 and 2 were aggregated as the Myanmar Artemisinin Resistance Containment (MARC) areas and were compared with tier 3 townships, which were defined as non-MARC areas. The chi-square test was used to compare groups, and the level of significance was set at *P* ≤ 0.05.

**Results:**

Of the 6328 households assessed, 97.2% in both MARC and non-MARC areas had at least one bed net (any type), but only 63% of households had ITNs/LLINs. Only 44% of households in MARC areas and 24% in non-MARC areas had adequate numbers of ITNs/LLINs (one ITN/LLIN per two persons, *P* <  0.001). Nearly 44% of household members had access to ITNs/LLINs. Regarding the utilization of ITNs/LLINs, 45% of household members used them in MARC areas and 36% used them in non-MARC areas (*P* <  0.001, desired target = 100%). Utilization of ITNs/LLINs among children aged below five years and pregnant women (high malaria risk groups) was low, at 44% and 42%, respectively.

**Conclusions:**

This study highlights the nationwide shortfalls in the ownership of, access to, and utilization of ITNs/LLINs in Myanmar, which is of particular concern in terms of containing the spread of artemisinin resistance. It highlights the need for priority attention to be paid and mobilization of resources in order to improve bed net coverage and utilization through bed net distribution and/or social marketing, information dissemination, and awareness-raising.

**Electronic supplementary material:**

The online version of this article (10.1186/s40249-018-0399-2) contains supplementary material, which is available to authorized users.

## Multilingual abstracts

Please see Additional file 1 for translations of the abstract into the five official working languages of the United Nations.

## Background

Malaria is a global public health problem with 212 million reported cases in 2015 and an estimated 429 000 deaths [1]. Myanmar in South-East Asia is one of the 31 high burden malaria countries in the world, with 240 000 reported cases in 2015 [1]. The country lies in the Greater Mekong Subregion (GMS) [2, 3], and drug efficacy studies have revealed *Plasmodium falciparum* resistance to artemisinin in Myanmar and four other countries in the GMS (Cambodia, the Lao People’s Democratic Republic, Thailand, and Vietnam) [2–4]. There is great concern that such resistance could spread in the region and beyond, thereby undoing gains made so far in global malaria control [3, 5].

A vital intervention for ensuring reduction of malaria transmission and eventual elimination of malaria, including resistant strains, is the use of insecticide-treated bed nets/long-lasting insecticidal nets (ITNs/LLINs) for indoor transmission [3, 6–8]. Bed nets reduce transmission of malaria through the mosquito vector, by interrupting transmission from humans to mosquitoes and then back to humans. It has been shown that ITNs reduce the incidence of uncomplicated malaria episodes by 50% in areas of stable malaria [7], and reduce malaria mortality in children by 55% in systematic literature review in *Plasmodium falciparum* endemic setting [8] and by 19–24% in Ghana study [6], respectively.

Myanmar aims at achieving and sustaining 100% access to and utilization of ITNs/LLINs at the household level and, as such, continued monitoring of these parameters is seen as priority operational research for the National Malaria Control Program (NMCP) [3, 9, 10]. This has been pertinent to Myanmar since it embarked on the Myanmar Artemisinin Resistance Containment (MARC) program [9]. The goals of MARC are to prevent the spread of artemisinin-resistant parasites within the country and beyond, and to reduce transmission, morbidity, and mortality of *P. falciparum* malaria, with priority given to areas threatened by artemisinin resistance [11].

Artemisinin resistance in the country is stratified as follows [2, 10]: tier 1 areas is where there is credible evidence of artemisinin resistance; tier 2 is where there is a significant inflow of people from tier 1, including those areas immediately bordering tier 1; and tier 3 is where there is no evidence of artemisinin resistance and limited contact with tier 1 areas. MARC areas encompass tier 1 and 2 areas, while non-MARC areas encompass tier 3.

Although two other published studies have reported on bed net coverage and utilization in Kachin State and Tanintharyi Region of Myanmar [12, 13], these studies did not assess coverage and utilization in relation to different areas with evidence of artemisinin resistance. The present study used national program data and covered the entire population residing in malaria-risk areas from all states/regions of Myanmar. It is thus representative of and adds value to program implementation under the National Malaria Strategic Plan (2016–2021).

This study thus aimed at examining household ownership of, access to, and utilization of bed nets in areas of Myanmar with and without artemisinin resistance containment measures (stratified into MARC and non-MARC areas). Specific objectives were to compare: a) household demographic characteristics; b) ownership of, access to, and utilization of bed nets; and c) characteristics of the bed nets, including their physical condition.

## Methods

### Study design

This was a retrospective analysis of secondary data from a nationwide community-based, cross-sectional malaria survey conducted in 2014.

### Study setting

The Republic of the Union of Myanmar has an estimated population of 51 million inhabitants [14]. The country is bordered by Bangladesh, India, China, Lao People’s Democratic Republic, and Thailand in the north and east, the Bay of Bengal in the west, and the Andaman Sea in the south. It is divided administratively into the capital territory (Nay Pyi Taw Council Territory), seven states and seven regions. There are 74 districts with 330 townships [9].

Malaria is endemic in 284 townships (86.1%, 284/330) of Myanmar. The country is divided into high transmission (> 1 case per 1000 population), low transmission (0–1 case per 1000 population), and malaria-free areas (zero cases); 15.8% are high transmission areas, 43.8% are low transmission areas, and 40.4% are malaria-free areas [1]. Regarding artemisinin resistance, 52 townships belong to tier 1, 20 to tier 2, and 258 to tier 3.

### Status of ITN/LLIN distribution and usage in the study setting

Free distribution of ITNs/LLINs in areas of high malaria transmission is one of the key interventions for malaria elimination in Myanmar. This is mainly done by the NMCP and other stakeholders, and is reserved for malaria endemic areas.

Although ITNs/LLINs are internationally recommended, other types of untreated bed nets made of cotton, nylon, and lace are also available from local markets. Efficacy is monitored on a yearly basis. If the nets are damaged, they should be replaced, and otherwise they should be replaced every three years, but this is not yet practiced. *Anopheles dirus* and *An. minimus* are the two primary vectors for malaria in Myanmar [15].

### Survey data

The data for this study were sourced from a community-based survey conducted by the NMCP and the Department of Medical Research (DMR) in 2014 in malaria-risk areas. The survey mainly addressed five issues: 1) social and demographic characteristics; 2) knowledge, attitudes, and practices towards prevention; 3) coverage and bed net use patterns; 4) treatment-seeking practices for fever; and 5) forest movement behaviors by the community. For this study, a subset of data on the coverage of ITNs/LLINs was extracted.

A multistage sampling procedure was used in this survey jointly conducted by the NMCP and the DMR. Thirty townships were randomly selected among 284 malaria endemic townships. At the township level, eight villages were randomly selected, and in each village 25–30 households were systematically selected using a pre-defined list. If a selected household did not exist, it was replaced with the next household in line. The pre-defined list of actual village households was updated by the survey teams’ supervisors with help from the village leaders. Among the selected households, 97.5% participated in the questionnaire interview.

In the selected households, face-to-face interviews (based on semi-structured questionnaires) were conducted with the, preferably, female adult respondent (as they were considered to be more likely to have the necessary information), or any adult in case a female adult was not available. Interviewers also observed the type (LLIN/ITN/ordinary) and condition (good/repaired/with holes) of each bed net after asking how many bed nets there were in the household.

Questionnaires were pre-tested, and all interviewers were trained in each state/region by the NMCP and the DMR. The interviewers were also trained on how to differentiate between LLINs and ITNs. All interviews were conducted in the Myanmar local language.

Survey data were double entered and validated using EpiData Entry software (version 3.1, EpiData Association, Odense, Denmark). The survey database is available from the NMCP, the Ministry of Health and Sports, Myanmar.

### Data variables and outcomes

The ownership of bed nets at the household level was assessed using two indicators: a) availability of at least one ITN/LLIN per household, and b) at least one ITN/LLIN per two household members [1].

Indicators for achieving universal access to and utilization of ITNs/LLINs included the proportion of household members with access to ITNs/LLINs in their households and proportion of household members who slept under an ITN/LLIN the previous night. Additional information on the physical condition of bed nets was also gathered using direct observation, and included material type and presence or absence of holes. For this study, tiers 1 and 2 were aggregated as the MARC area and compared with tier 3, which was defined as the non-MARC area.

### Statistical analysis

Data related to bed nets were extracted from the main survey database, exported from the EpiData database and imported to Stata (version 11, StataCorp, TX, USA) for analysis.

We used frequency and proportion(s) to summarize the baseline characteristics and study outcomes. The chi-square test was used to make comparisons between MARC and non-MARC areas, with the level of significance set at *P* ≤ 0.05.

The below indicators, which are related to the study’s objectives, were calculated following Household Survey Indicators for Malaria Control guidelines [16]:

- *Proportion of households with at least one ITN/LLIN*
  - Numerator: Number of households surveyed with at least one ITN/LLIN.
  - Denominator: Total number of households surveyed
- *Proportion of households with at least one ITN/LLIN for every two people*
  - Numerator: Number of households surveyed with at least one ITN/LLIN for every two people.
  - Denominator: Total number of households surveyed
- *Proportion of the population with access to an ITN/LLIN in their household*
  - Numerator: Total number of individuals who can sleep under an ITN/LLIN if each ITN/LLIN in the household is used by two people.
  - Denominator: Total number of individuals who spent the previous night in the surveyed households
- *Proportion of the population that slept under an ITN/LLIN the previous night*
  - Numerator: Number of individuals who slept under an ITN/LLIN the previous night.
  - Denominator: Total number of individuals who spent the previous night in the surveyed households.

## Results

### Characteristics of the study population and household demographic characteristics

Of the 6490 households involved in the community-based survey, 6328 (97.5%) completed the interview. Household demographic characteristics such as gender and age group were similar in MARC and non-MARC areas (see Table 1).

**Table 1:** Demographic characteristics of household members assessed for ownership, access and utilization of bed nets stratified by Myanmar Artemisinin Containment Areas (MARC) and non-MARC areas of Myanmar; 2014

Demographic characteristics	Total *n* (%)	MARC area *n* (%)	Non MARC area *n* (%)
Households assessed	6328	1714 (27.1)	4614 (72.9)
Household members	31 626	8509 (26.9)	23 117 (73.1)
Gender			
Male	15 253 (48.2)	4193 (49.3)	11 060 (47.8)
Female	16 373 (51.8)	4316 (50.7)	12 057 (52.2)
Age group (years)			
< 5	2713 (8.6)	835 (9.8)	1878 (8.1)
5–15	7684 (24.3)	2182 (25.6)	5502 (23.8)
16–59	18 474 (58.4)	4747(55.8)	13 727 (59.4)
≥ 60	2755 (8.7)	745 (8.8)	2010 (8.7)

### Household ownership of, access to, and utilization of bed nets

Table 2 shows household ownership of, access to, and utilization of bed nets stratified by MARC and non-MARC areas.

**Table 2:** Household ownership, access and utilization of bed nets stratified by Myanmar Artemisinin Containment Areas (MARC) and non-MARC areas of Myanmar, 2014

		Artemisinin resistance areas		
	Total	MARC	Non-MARC	*P-*value
	*n* (%)	*n* (%)	*n* (%)	
Household ownership of bed nets				
Total households	6328	1714	4614	
At least one net per household (any type)	6153 (97.2)	1694 (98.8)	4459 (96.6)	< 0.001
At least one ITN/LLIN per household	4007 (63.3)	1325 (77.3)	2682 (58.1)	< 0.001
One net per two people (any type)	4145 (65.5)	1223 (71.4)	2922 (63.3)	< 0.001
One ITN/LLIN per two people	1878 (29.7)	757 (44.2)	1121 (24.3)	< 0.001
Access and utilization of ITN/LLIN				
Total number of household members^a^	28 876	7832	21 044	
% of population with access to an ITN/LLIN	13 569 (43.5)	4810 (61.4)	8759 (41.6)	< 0.001
% of population who slept under an ITN/LLIN	11 204 (38.8)	3555 (45.4)	7649 (36.4)	< 0.001

^a^Includes individuals who slept in the household the previous night

While almost all households (97.2%) had at least one bed net, only 63% had ITNs/LLINs. Similarly, only about 30% of all households had sufficient numbers of ITNs/LLINs to meet the desired target of at least one ITN/LLIN per two persons in the household. Only 44% of households in MARC areas and 24% in non-MARC areas had adequate numbers of ITNs/LLINs (one ITN/LLIN per two persons, *P* <  0.001).

Nearly 44% of household members had access to ITNs/LLINs, but only 39% slept under ITNs/LLINs during the previous night. Regarding the utilization of ITNs/LLINs, 45% of household members used them in MARC areas and 36% used them in non-MARC areas (*P* <  0.001, desired target = 100%).The proportion of children under five years (*n* = 2713) sleeping under an ITN/LLIN the previous night was 44%, while among pregnant women (*n* = 238) it was 42%.

### Characteristics of bed nets, including their physical condition

Table 3 shows the characteristics of surveyed bed nets at the household level.

**Table 3:** Characteristics of bed nets in households stratified by Myanmar Artemisinin Containment Areas (MARC) and non-MARC areas of Myanmar, 2014

		Artemisinin resistance areas		*P-*value
Characteristics	Total	MARC	Non-MARC	
	*n* (%)	*n* (%)	*n* (%)	
Total bed nets	17 698	5446	12 252	
Bed net type (*N* = 17 583)				
Non-LLIN	11 935 (67.9)	2861 (52.8)	9074 (74.6)	< 0.001
LLIN	5648 (32.1)	2554 (47.2)	3094 (25.4)	
Bed net size (*N* = 17 546)				
One person size	1780 (10.1)	391 (7.2)	1389 (11.5)	< 0.001
One and half person size	739 (4.2)	151 (2.8)	588 (4.9)	
Two persons size	7552 (43.0)	3312 (61.2)	4240 (34.9)	
Family size	7475 (42.6)	1557 (28.8)	5918 (48.8)	
Bed net condition (*N* = 17 216)				
Good (No. holes)	13 543 (78.7)	4441 (83.0)	9102 (76.7)	< 0.001
Repaired (No. holes)	953 (5.5)	213 (4.0)	740 (6.2)	
Holes	2720 (15.8)	699 (13.1)	2021 (17.0)	
Insecticide treatment status (*N* = 17 420)				
Untreated (Ordinary net)	8557 (49.1)	1949 (36.2)	6608 (54.9)	< 0.001
LLIN	3934 (22.6)	2139 (39.7)	1795 (14.9)	
ITN	4929 (28.3)	1302 (24.2)	3627 (30.2)	
Time since last insecticide treatment^a^ (*N* = 4755)				
Less than 6 months	1026 (21.6)	332 (26.4)	694 (19.9)	< 0.001
6 months to 1 year	2036 (42.8)	424 (33.7)	1612 .1)	
> 1 year	1693 (35.6)	503 (40.0)	1190 (34.0)	

Missing omitted ITN - Insecticide treated bed nets, *LLIN* Long lasting insecticidal nets

^a^Only applicable to ITN

Less than half of all bed nets were ITNs/LLINs. The main sources of LLINs (the most desired type of bed net) were government (63%) and non-governmental organizations (NGOs) (31%). Of all nets, 21% had holes or had already undergone repairs. The proportion of bed nets with holes was 13% in MARC areas and 17% in non-MARC areas (*P* <  0.001). Regarding the insecticide treatment status, an estimated 49% were untreated bed nets. Among the ITNs, only 22% were within the desired expiry date of six months. There were more effective ITNs in MARC areas (26.4%) than in non-MARC areas (19.9%) (*P* <  0.001).

## Discussion

This is the first nationwide study assessing household ownership of, access to, and utilization of bed nets in areas with and without artemisinin resistance containment measures in Myanmar. The study found that 63% households had at least one ITN/LLIN, but only 30% had sufficient ITNs/LLINs. Although these figures are lower than desired, they do show an improvement in relation to what was the case in 2011 [17]. There was an increase in household ownership of at least one ITN/LLIN from 11% in 2011 to 77% in 2014 due to mass distribution of treated nets by The Global Fund to Fight AIDS, Tuberculosis and Malaria (four million) and the President’s Malaria Initiative (90000). Similarly, sufficient ITNs/LLINs at the household level increased from 3% in 2011 to 44% in 2014 in MARC areas. The proportion of household members who slept under an ITN/LLIN the previous night also increased from 18% in 2011 to 45% in 2014 in MARC areas. However, the number of treated nets distributed in Myanmar is still disproportionate to the estimated population of 38 million living in malaria-endemic regions of the country. Further community engagement activities are essential to motivate households to use LLINs more than they currently do. There is also a need to strengthen replacement strategies to ensure sustained use of effective ITNs/LLINs in targeted areas.

Despite improvements being made since the previous survey conducted in 2011, the findings from the 2014 study serve as a wake-up call to reinforce collaborative efforts and to seek additional resources to improve the state of affairs related to household survey indicators for malaria control. This is of particular relevance in the GMS, where frequent population movements are known to occur [3]. Populations seeking better income and other opportunities move about seasonally and can miss the chance to receive LLINs distributed by malaria control programs. Effective collaboration between the government and international NGOs from neighboring countries is thus essential for control of population movement leading towards malaria control and elimination [18].

This study’s strengths are that data were sourced from a nationwide survey and are thus likely to be a true reflection of the situation on the ground. We also adhered to Strengthening the Reporting of Observational Studies in Epidemiology guidelines for the reporting of observational studies [19]. In addition, data accuracy was validated with results from a recent Myanmar Demographic and Health Survey [20], in which 97% of the 13 260 surveyed households were found to own any mosquito net and only 21% of household members were found to have access to an ITN. This result was more or less similar to the 24% of household members having access to an ITN in non-MARC areas as found in the present study.

The study findings have a number of policy and practice implications. First, although almost all households owned bed nets, only 63% were ITNs/LLINs, the recommended type. Even in MARC areas, sufficient ITN/LLIN ownership (one ITN/LLIN per two persons) was far below the desired target of 100%. This suggests the need for more frequent bed net distribution coupled with knowledge and awareness-raising to improve the utilization of ITNs/LLINs. The observed shortages in household availability may also be partly due to usage for other purposes and/or sale of bed nets for additional revenue. As LLINs have been shown to be more effective and long lasting (up to three years) than ordinary nets [21, 22], there is an urgent need to improve LLIN procurement and distribution. Possible ways forward include mass community distribution campaigns and/or social marketing strategies.

Second, even when households had access to ITNs/LLINs, a considerable proportion did not use them. This has already been observed in other studies conducted in Nigeria, Kenya, and Tanzania [23–25], and may be related to the lack of knowledge on the importance of ITNs/LLINs or perceived problems of ITNs/LLINs such as insecticide smell, and their ability to cause dizziness, headaches, or difficulty breathing. This has been reported among migrant workers in Myanmar [10]. The regular use of bed nets can significantly reduce the occurrence of malaria infection as seen in a study conduced in India [26]. Further qualitative research may be useful to determine the main drivers or barriers of ITN/LLIN utilization and to aid targeted behavior change communication strategies.

Third, the majority of available ITNs were considered ineffective in terms of insecticide impregnation due to their untreated nature or expiration of the insecticide effect. Entomological studies that verify the effectiveness of ITNs would seem justified to better understand the residual efficacy of the insecticides used for treated nets. The best practical option is to ensure the rapid transition to LLINs in all areas of Myanmar.

Finally, less than half of those at particular risk of malaria (children under five years and pregnant women) were found to have slept under an ITN/LLIN the previous night. There is no targeted strategy to distribute LLINs to high-risk groups in Myanmar. A major reason is the NMCP staff not specifically listing households with children and pregnant women before distributing LLINs. The NMCP’s overall target for the number of LLINs distributed to at-risk households in endemic regions should be one LLIN per two people [27]. The minimum coverage of LLINs (at least 80%) for vulnerable groups is not achieved. This is of particular concern as malaria-related morbidity and mortality is the highest in these groups [1]. In Myanmar, the NMCP needs to prioritize these subgroups to ensure they receive 100% access to ITNs/LLINs.

## Conclusions

This study highlights nationwide shortfalls in household ownership of, access to, and utilization of ITNs/LLINs in Myanmar. This underlines the need for priority attention to be paid and mobilization of resources in order to improve bed net coverage and utilization through bed net distribution and/or social marketing, information dissemination, and awareness-raising.

## Additional file

Additional file 1: Multilingual abstracts in the five official working languages of the United Nations. (PDF 1024 kb)

## Abbreviations

DMR: Department of Medical Research
GMS: Greater Mekong Subregion
ITN: Insecticide-treated bed net
LLIN: Long-lasting insecticidal net
MARC: Myanmar Artemisinin Resistance Containment
NGO: Non-governmental organization
NMCP: National Malaria Control Program
