# Supplementary material for: Assessment of household ownership of bed nets in areas with and without artemisinin resistance containment measures in Myanmar
Source: Infect Dis Poverty. 2018 Mar 23;7:19. doi: 10.1186/s40249-018-0399-2 (PMC5865351; doi:10.1186/s40249-018-0399-2)
Supplement: Supplementary file 1 — Multilingual abstracts in the five official working languages of the United Nations. (PDF 1024 kb) [file 40249_2018_399_MOESM1_ESM.pdf]

Translation of the abstract into the five official working languages of the United Nations

تدابير الاحتواء في المناطق التي بها والتي ليس بها مقاومة لمادة الأرتيميسينين في ميانمارعن طريق اقتناء والوصول إلى واستخدام الناموسيات بين المهاجرين الدائمين والموسميين

ثاي ماونج ماونج، تين أو، خين ثيت واي، ثونج هلاينج، فيليب أويتي، بيناي كومار، هيمانت شيواد،روني زكريا، أونج ثي

الملخص

خلفية: تقع ميانمار في الإقليم الفرعي ميكونج الكبرى الذي يواجه التحدي المتمثل في المقاومة الناشئةللعلاجات المركبة المكونة أساسًا من مادة الأرتيميسينين (ACT). وبما أن مركب الأرتيميسينين هو الركيزة الأساسية للعلاجات الفعالة المضادة للملاريا، فإن احتواء مقاومة الأرتيميسينين هو أولوية وطنية وعالمية. ويعتبر استخدام الناموسيات المعالجة بالمبيدات الحشرية / الناموسيات الطويلة الأمد هو التدخل الرئيسي لضمان الحد من انتقال الملاريا وانتشار السلالات المقاومة، والقضاء على الملاريا في نهاية المطاف. تهدف الدراسة إلى تقييم تدابير الاحتواء في المناطق التي بها والتي ليس بها مقاومة لمادة الأرتيميسينين في ميانمارعن طريق اقتناء والوصول إلى واستخدام الناموسيات بين المهاجرين الدائمين والموسميين
الأساليب: وجرى تحليل البيانات الثانوية المستمدة من دراسة استقصائية مجتمعية وطنية لمكافحة الملاريا أجراها البرنامج الوطني لمكافحة الملاريا في عام 2014. واستنادا إلى الأدلة على مقاومة الأرتيميسينين، تم تقسيم ميانمار إلى مستويات 1 و 2 و 3: تم تجميع البلدات في المستويين 1 و 2 باعتبارها مناطق ميانمار الواقعة في مجال مقاومة الأرتيميسينين وتمت مقارنتها بلدات المستوى 3، المناطق غير الواقعة في مجال مقاومة الأرتيميسينين. تم استخدام اختبار مربع كاي لمقارنة المجموعات، وتم تعيين مستوى أهمية عند ≥P.0.05 ≥0.05.
النتائج: من بين 6328 أسرة تم تقييمها، كان 97,2٪ في كل من مناطق ميانمار الواقعة في مجال مقاومة الأرتيميسينين والمناطق غير الواقعة في مجال مقاومة الأرتيميسينين لديها ناموسية واحدة على الأقل (أي نوع)، ولكن 63٪ فقط من الأسر لديها ناموسيات معالجة بالمبيدات الحشرية / ناموسيات طويلة الأمد. نسبة 44% فقط من الأسر في المناطق مناطق ميانمار الواقعة في مجال مقاومة الأرتيميسينين و 24٪ في المناطق غير الواقعة في مجال مقاومة الأرتيميسينين كان لديها أعداد كافية من الناموسيات المعالجة بالمبيدات الحشرية/الناموسيات طويلة الأمد (ناموسية واحدة لكل شخصين،P (0.001>) وكان ما يقرب من 44% من أفراد الأسر لديهم ناموسيات معالجة بالمبيدات الحشرية / ناموسيات طويلة الأمد. وفيما يتعلق باستعمال الناموسيات المعالجة بالمبيدات الحشرية، استخدم 45 % من أفراد الأسر في مناطق ميانمار الواقعة في مجال مقاومة الأرتيميسينين وبينما كانت النسبة 36% في المناطق غير الواقعة في مجال مقاومة الأرتيميسينين P <0.001، الهدف المطلوب = 100%). وكان استخدام الناموسيات المعالجة بالمبيدات الحشرية / الناموسيات طويلة الأمد بين الأطفال الذين تقل أعمارهم عن خمس سنوات والنساء الحوامل (مجموعات درجة مخاطر تعرضها لملاريا عالية) منخفضا، حيث بلغ 44% و 42% على التوالي.
الاستنتاجات:هذه الدراسة تسلط الضوء على أوجه القصور الوطنية في اقتناء، والوصول إلى واستخدام الناموسيات المعالجة بمبيدات الحشرات / الناموسيات طويلة الأمد في ميانمار، والتي هي ذات أهمية خاصة في احتواء انتشار مقاومة الأرتيميسينين. وتركز على ضرورة الاهتمام بإعطاء الأولوية وتعبئة الموارد من أجل نشر استخدام الناموسيات من خلال التوزيع و / أو التسويق الاجتماعي، ونشر المعلومات، وزيادة الوعي.

Translated from English version into Arabic by Mahmoud Sami, proofread by Nancy Muhammed, through

TRANSLATORS
WITHOUT BORDERS

缅甸青蒿素抗性地区蚊帐的家庭覆盖率、获取及利用情况

Thae Maung Maung, Tin Oo, Khin Thet Wai, Thaung Hlaing, Philip Owiti, Binay Kumar, Hemant Shewade, Rony Zachariah, Aung Thi

摘要

引言：缅甸位于大湄公河次区域，在此区域恶性疟原虫对青蒿素出现了抗性。由于“青蒿素”化合物是有效抗疟疾治疗的支柱，因此抑制青蒿素耐药性的传播成为国家和全球的首要任务之一。使用经杀虫剂处理的蚊帐/长效杀虫蚊帐（ITN/LLIN）是确保减少疟疾和耐药虫株传播，以及消除疟疾的关键干预措施。本研究旨在评估缅甸青蒿素抗性地区蚊帐的获取和使用情况。
方法：对 2014 年全国疟疾防治规划进行的全国性社区疟疾调查的二手资料进行分析。根据青蒿素抗性的证据，将缅甸划分为 1、2、3 级区域。在本研究中，将第 1 级和第 2 级合并为缅甸青蒿素抗性遏制（MARC）区域，并将第 3 级区域划分为非青蒿素抗性遏制区域。采用卡方检验对各组进行比较，显著性水平定为 P≤0.05。
结果：MARC 和非 MARC 地区的 6 328 个评估家庭中，≥97%的家庭至少拥有一顶蚊帐（任何类型），但仅 63%的家庭拥有 ITN/LLIN。MARC 和非 MARC 地区分别只有 44%和 24%的家庭有足够数量的 ITN/LLIN（每两人一顶 ITN/LLIN，*P*<0.001）。近 44%的家庭成员可使用 ITN/LLIN。ITN/LLIN 的利用率在 MARC 地区为 45%，非 MARC 地区为 36%（*P*<0.001，预期目标=100%）。5 岁以下儿童和孕妇（高疟疾风险群体）的 ITN/LLIN 使用率较低，分别为 44%和 42%。
结论：本研究突出表明了全国范围内 ITN/LLIN 的覆盖率、获取及利用方面的不足，这对于抑制青蒿素耐药性来说尤其令人担忧。这说明需要优先关注并调动资源，通过蚊帐分发和/或社会营销、信息及教

育意识的提高来改善蚊帐的覆盖率和利用率。

Translated from English version into Chinese by Translated by Xue-Jiao Ma, edited by Pin Yang

Propriété par les ménages, leur accès et leur utilisation des moustiquaires de lit dans les régions du Myanmar qui ont appliqué ou non des mesures de contrôle de résistance à l'artémisinine

Thae Maung Maung, Tin Oo, Khin Thet Wai, Thaung Hlaing, Philip Owiti, Binay Kumar, Hemant Shewade, Rony Zachariah, Aung Thi

Résumé

Contexte: Le Myanmar se trouve dans la sous-région du Grand Mékong, là où il y a de la résistance au *Plasmodium falciparum*et à l'artémisinine. Comme le composé d'artémisinine est au centre des thérapies efficaces contre le paludisme, la contrôle de la propagation de la résistance à l'artémisinine représente une priorité nationale et mondiale. L'utilisation de moustiquaires traitées à l'insecticide pour et de moustiquaires imprégnées d'insecticides de longue durée (MTI/MII) est primordiale pour garantir la réduction de la propagation du paludisme et de souches bactériennes résistantes et, éventuellement, éradiquer le paludisme. Cette étude vise à évaluer la propriété par les ménages, leur accès et leur utilisation de moustiquaires de lit dans les régions du Myanmar qui ont appliqué ou non des mesures de contrôle de résistance à l'artémisinine.

Méthodes: Des données secondaires d'enquêtes communautaires menées à l'échelle nationale par le Programme national de contrôle du paludisme en 2014 ont été analysées. Selon les données probantes sur la résistance à l'artémisinine, le Myanmar a été divisé en trois niveaux (1, 2, 3) : les cantons des niveaux 1 et 2 ont été agrégés en régions MARC (contention de la résistance à l'artémisinine du Myanmar) avec les cantons de niveau 3 qui ont été déclarés comme régions non-MARC. Un test du chi carré a été utilisé pour comparer les groupes et le niveau d'importance a été établi à $P \leq 0{,}05$.

Résultats: Sur les 6 328 ménages évalués, 97,2 % des ménages des régions MARC et non-MARC disposaient d'au moins une moustiquaire de lit (tous types confondus), mais seuls 63 % des ménages disposaient de MTI/MII de longue durée. Seuls 44 % des foyers des régions MARC et 24 % des régions non-MARC disposaient de suffisamment de MTI/MII de longue durée (une pour deux personnes, $P < 0{,}001$). Il est estimé que 44 % des membres des ménages avaient accès aux MTI/MII de longue durée. Pour ce qui concerne les MTI/MII de longue durée, 45 % des membres des ménages s'en servaient dans les régions MARC, contre 36 % pour les régions non-MARC ($P < 0{,}001$, cible souhaitée = 100 %). L'utilisation de MTI/MII de longue durée parmi les enfants de moins de cinq ans et les femmes enceintes (des groupes à fort risque de paludisme) était faible, à 44 % et 42 %, respectivement.

Conclusions: Cette étude révèle des lacunes importantes à l'échelle national, dans la propriété, l'utilisation, l'accès et l'utilisation des MTI/MII au Myanmar, ce qui s'avère particulièrement problématique pour le contrôle de la propagation de résistance à l'artémisinine. Elle révèle la nécessité de porter une attention prioritaire et de mobiliser les ressources afin d'améliorer les données relatives aux moustiquaires de lit et leur utilisation par une distribution des moustiquaires de lit et/ou le marketing social, la diffusion de renseignements et la sensibilisation.

Translated from English version into French by J-Gabriel, proofread by Nellie K. Adaba, through

Наличие в собственности домохозяйств надкроватных сеток, доступ к ним и их использование в областях Мьянмы, где проводятся и не проводятся меры по сдерживанию резистентности к артемизинину

Таэ Маунг Маунг, Тинь У, Кхин Тхет Вай, Таунг Хлаинг, Филип Оуити, Винай Кумар, Хемант Шевейд, Рони Захариа, Аунг Тхи

Аннотация

Вводные данные: Мьянма находится в субрегионе Большого Меконга Юго-Восточной Азии, где существует резистетность *Plasmodium falciparum* к артемизинину. Поскольку препарат, содержащий артемизинин, является основой эффективной противомалярийной терапии, сдерживание распространения резистентности к артемизинину представляет собой приоритетную задачу на национальным и глобальным уровне. Использование обработанных инсектицидами надкроватных сеток/инсектицидных сеток длительного использования (ИНС/ИСДИ) является основной мерой, направленной на уменьшение передачи малярии и распространения резистентных штаммов с целью окончательной ликвидации малярии. Данное исследование посвящено изучению наличия в собственности домохозяйств надкроватных сеток, доступа к ним и их использованию в областях Мьянмы, где проводятся или не проводятся меры по сдерживанию резистентности к артемизинину.
Методы: Были проанализированы вторичные данные опроса общин, проведенного на национальном уровне в рамках Национальной программы контроля малярии в 2014 году. Исходя из данных резистентности к артемизинину, Мьянму поделили на уровни 1, 2 и 3: поселения уровней 1 и 2 были объединены в области сдерживания резистентности к артемизинину в Мьянме (Myanmar Artemisinin Resistance Containment, MARC) и сравнивались с поселениями уровня 3, которые были определены как не относящиеся к MARC. Для сравнения групп использовался критерий хи-квадрат, а уровень значимости был определен как $P \leq 0{,}05$.
Результаты: Из 6328 оцененных домохозяйств 97,2 % в поселениях, как относящихся, так и не относящихся к MARC, имели как минимум одну надкроватную сетку (любого типа), но только 63 % домохозяйств имели ИНС/ИСДИ. Лишь 44 % домохозяйств в районах MARC и 24 % в районах, не относящихся к MARC, имели достаточное количество ИНС/ИСДИ (одна ИНС/ИСДИ на двух человек, $P < 0{,}001$). Около 44 % членов домохозяйства имели доступ к ИНС/ИСДИ. Что касается использования ИНС/ИСДИ, ими пользовались 45 % членов домохозяйств в районах MARC и 36 % членов домохозяйств в районах, не относящихся к MARC ($P < 0{,}001$, желательная цель — 100 %). Использование ИНС/ИСДИ среди детей в возрасте до пяти лет и беременных женщин (группы высокого риска малярии) было небольшим: 44 % и 42 % соответственно.
Выводы: В этом исследовании освещаются общенациональные недостатки, связанные с владением, доступом и использованием ИНС/ИСДИ в Мьянме, которые вызывают большую озабоченность в контексте сдерживания распространения резистентности к артемизинину. В нем подчеркивается необходимость концентрации внимания и мобилизации ресурсов для улучшения охвата населения и повышения использования надкроватных сеток посредством дистрибуции и/или социального маркетинга, распространения информации и повышения осведомленности о надкроватных сетках.

Translated from English version into Russian by Oksana Weiss, proofread by Liudmila Tomanek, through

TRANSLATORS WITHOUT BORDERS

Propiedad, acceso y utilización de mosquiteros en áreas de Myanmar con o sin medidas de contención de resistencia a la artemisinina

Thae Maung Maung, Tin Oo, Khin Thet Wai, Thaung Hlaing, Philip Owiti, Binay Kumar, Hemant Shewade, Rony Zachariah, Aung Thi

Resumen

Trasfondo: Myanmar se encuentra en la subregión del Gran Mekong donde hay *Plasmodium falciparum* resistencia a la artemisinina. Como el compuesto de artemisinina es el pilar de las terapias efectivas contra la malaria, contener el avance de la resistencia a la artemisinina es una prioridad nacional y global. El uso de mosquiteros tratados con insecticida/mosquiteros insecticidas de larga duración (ITN/LLIN, por sus siglas en inglés) es la intervención clave para reducir la transmisión de la malaria y la propagación de cepas resistentes, y para eventualmente, lograr eliminar la malaria. Este estudio tenía como objetivo evaluar la propiedad, acceso y utilización de mosquiteros en áreas de Myanmar con o sin medidas de contención de resistencia a la artemisinina.
Métodos: se analizaron los datos secundarios de una encuesta de malaria realizada por todo el país, a nivel comunitario, gestionada por el Programa Nacional de Control de Malaria en 2014. Basándose en evidencia de resistencia a la artemisinina, Myanmar fue dividido en niveles 1, 2 y 3: los municipios de niveles 1 y 2 se agruparon como áreas de Contención de Resistencia a la Artemisinina de Myanmar (MARC por sus siglas en inglés) y se compararon con los municipios de nivel 3, que se definieron como áreas no MARC. La prueba de chi-cuadrado se utilizó para comparar grupos, y el nivel de importancia se estableció en $P \leq 0.05$.

Resultados: de los 6,328 hogares evaluados, el 97.2% en las áreas MARC y no MARC tenían al menos un mosquitero (de cualquier tipo), pero solo el 63% de los hogares tenían ITN/LLIN. Solo el 44% de los hogares en áreas MARC y el 24% en áreas no MARC tenían un número adecuado de ITN/LLIN (un ITN/LLIN por cada dos personas, $P < 0.001$). Casi el 44% de los miembros del hogar tenían acceso a ITN/LLIN. Con respecto a la utilización de ITN/LLIN, el 45% de los miembros del hogar los usaron en áreas MARC y el 36% los usaron en áreas no MARC ($P < 0.001$, meta deseada = 100%). La utilización de ITN/LLIN entre los menores de cinco años y las mujeres embarazadas (grupos con alto riesgo de malaria) fue baja, con un 44% y un 42%, respectivamente.
Conclusiones: este estudio revela importantes deficiencias nacionales de propiedad, acceso y utilización de ITN/LLIN en Myanmar, lo que es especialmente preocupante en términos de contención de la propagación de la resistencia a la artemisinina. Resalta la necesidad de prestar atención prioritaria y movilizar recursos para mejorar la protección con mosquiteros y su utilización mediante el reparto de mosquiteros y/o promoción social, difusión de información y sensibilización.

Translated from English version into Spanish by Amparo Muñoz, proofread by imcosa, through

TRANSLATORS
WITHOUT BORDERS
